# Dementia service readiness in Pakistan: a provincial health systems assessment using a novel WHO-AIMS aligned framework

**DOI:** 10.3389/fpubh.2026.1837649

**Published:** 2026-06-29

**Authors:** Syed Ali Bokhari, Tarik Qassem, Syed Fahad Javaid

**Affiliations:** 1 Al Amal Psychiatric Hospital, Emirates Health Services, Dubai, United Arab Emirates; 2 Maudsley Health, Dubai, United Arab Emirates; 3 Mohammed Bin Rashid University of Medicine and Health Sciences, Dubai, United Arab Emirates; 4 Department of Psychiatry, College of Medicine and Health Sciences, United Arab Emirates University, Al Ain, United Arab Emirates

**Keywords:** community mental health services, dementia, geriatrics, health policy, health services for the aged, Pakistan

## Abstract

**Background:**

Pakistan faces a rapidly growing dementia crisis with an estimated 374,060 cases, projected to rise substantially as the older adult population triples to 38.3 million by 2050. Yet no provincial-level assessment of service readiness has been published. To our knowledge, this study provides the first structured literature-based provincial assessment of dementia-relevant geriatric mental health infrastructure across Pakistan’s four provinces and two administrative territories.

**Methods:**

We conducted an integrative review synthesizing peer-reviewed literature, grey literature, and government reports. Drawing on the conceptual structure of WHO-AIMS (2007), we developed a novel six-criteria framework to categorize provincial readiness across three dimensions: governance, infrastructure, and specialized care. Findings were contextualized through comparison with India, Bangladesh, and Sri Lanka.

**Results:**

Our framework stratified Pakistan into three tiers. Tier 1 provinces (Punjab, Sindh) demonstrated partial system maturity; Tier 2 provinces (Khyber Pakhtunkhwa, Balochistan) exhibited foundational but inactive governance structures; and Tier 3 territories (Gilgit-Baltistan, Azad Jammu and Kashmir) showed absent legislative frameworks and no identifiable dementia services. Validated Urdu screening instruments exist (MMSE-Urdu, 10/66 battery) but are not systematically used. Pakistan’s psychiatric workforce (0.24 per 100,000) lags significantly behind regional comparators such as Sri Lanka (0.47) and India (0.30).

**Conclusion:**

Dementia care in Pakistan remains structurally dependent on urban tertiary centers in Punjab and Sindh; four of six jurisdictions have no identifiable dedicated dementia-specific services. Post-18th Amendment devolution created coordination gaps without capacity transfer. We propose a tiered implementation roadmap and multi-channel service delivery model leveraging primary care, community health workers, and telemedicine to bridge the specialist gap.

## 1 Introduction

Pakistan is undergoing a rapid demographic shift; its geriatric population, currently 11.1 million, is projected to more than triple to 38.3 million by 2050 (1). This demographic transition is expected to intensify pressure on health and long-term care systems, particularly where service infrastructure is already limited (2). Data from the Global Burden of Disease Study 2021 reveal that while age-standardized dementia prevalence rates have declined slightly (from 446.5 to 437.1 per 100,000), the absolute number of estimated cases has risen by approximately 82% since 1990 due to population aging (3). Crucially, this period has witnessed a significant rise in age-standardized mortality, suggesting that while incidence may be stabilizing, survival outcomes are worsening due to inadequate care (3). This burden is disproportionately borne by older females and compounded by high rates of metabolic risk factors and traumatic brain injury in the region (2–5). With an estimated 90% under-diagnosis rate, the true scale of this mortality likely far exceeds official estimates (3, 4, 6).

Pakistan’s capacity to respond to this burden was profoundly reshaped by the 18th Constitutional Amendment, which devolved health to provincial authorities and created a governance landscape marked by wide inter-provincial variation in implementation capacity (7, 8). Although devolution was intended to localize decision-making, the absence of strong federal coordination has contributed to a persistent gap between legislation and operational service delivery. Workforce density remains critically low, with recent national consultations estimating only 0.24 psychiatrists per 100,000 population, alongside severe urban concentration and the absence of a recognized geriatric psychiatry subspecialty pathway (6, 9). Provincial mental health authorities have been notified in several provinces, but their functionality varies sharply, leaving dementia care dependent on a small number of urban tertiary institutions rather than a coherent national or provincial system (6).

To facilitate future planning, this review adopts a multi-dimensional approach. Drawing on the conceptual structure of the World Health Organization Assessment Instrument for Mental Health Systems (WHO-AIMS), it develops a six-criteria provincial readiness framework to systematically categorize service availability across governance, infrastructure, and specialized care domains (10). It further synthesizes indigenous evidence, including validated screening instruments and cultural determinants of care, and situates Pakistan’s position within a regional comparative context (India, Bangladesh, and Sri Lanka) to highlight structural divergences in system organization. Accordingly, this study develops and applies the provincial readiness framework to map dementia service capacity across Pakistan, identify structural disparities, and propose a tiered implementation roadmap.

## 2 Methods

### 2.1 Study design

This study employs a comprehensive integrative review design, conducted in accordance with the updated integrative review methodology proposed by Whittemore and Knafl (11), specifically selected to synthesize heterogeneous evidence sources in a resource-limited setting.

We did not structure the review according to the Preferred Reporting Items for Systematic Reviews and Meta-Analyses (PRISMA) because the objective was an integrative, policy-facing synthesis of heterogeneous peer-reviewed, grey-literature, legislative, institutional, and service-mapping sources, rather than a narrowly reproducible systematic review of a defined intervention, exposure, diagnostic question, or effect estimate. We therefore used the integrative review framework of Whittemore and Knafl while providing the search strategy, eligibility criteria, grey-literature sources, and screening summary in Supplementary Table S2 (12).

To contextualize these findings, we conducted a structured comparative analysis benchmarking Pakistan against India, Bangladesh, and Sri Lanka. These comparators were purposively selected as they represent distinct governance archetypes evolving from a shared British colonial legal foundation. While Pakistan, India, and Bangladesh inherited the Indian Lunacy Act of 1912, Sri Lanka operates under the parallel Lunacy Ordinance of 1873, offering a valuable comparison of how similar colonial-era custodial frameworks have diverged under federal versus devolved governance. Comparative data were drawn from the WHO Mental Health Atlas (2020), WHO Global Dementia Observatory, and the STRiDE project (13–18).

### 2.2 Search strategy and data sources

We conducted a comprehensive search of electronic databases (PubMed, Scopus, and Google Scholar) between October and December 2025, with no date restrictions. To capture the full spectrum of the health system, search terms combined concepts related to dementia, geriatric psychiatry, memory clinics, provincial mental health legislation, health systems, and specific province or territory names (Punjab, Sindh, Khyber Pakhtunkhwa, Balochistan, Gilgit-Baltistan, and Azad Jammu and Kashmir). Searches were supplemented through targeted review of government portals, hospital and organizational websites, policy documents, and citation chaining from eligible sources (Supplementary Table S1).

To ensure a holistic assessment, we applied inclusion criteria covering clinical studies, policy documents, and grey literature. We specifically reviewed reports from international agencies, provincial laws, institutional service profiles, and publications relating to validated cognitive screening instruments, specialist service provision, and implementation barriers. Full details of the search strategy, including database-specific search strings, last-search dates, inclusion and exclusion criteria, grey literature identification methods, and a screening flow summary (Supplementary Figure S1) documenting database retrieval counts and final included source counts, are provided in Supplementary Table S2.

### 2.3 Data synthesis and quality assessment

Given the diverse nature of included sources, spanning epidemiological surveys, policy acts, and qualitative reports, formal systematic quality assessment tools were not employed, nor was a meta-analysis conducted. Instead, findings were synthesized narratively and organized thematically to map the service ecosystem. A supplementary table cataloging each included source by type, geography, date, and relevance to the analytical framework domains is provided in Supplementary Table S1. To ensure rigorous analysis despite these limitations, we developed and applied a novel structural framework to standardize the assessment of provincial capacities.

### 2.4 Data triangulation and validation

To enhance the rigor and validity of our findings, two reviewers independently applied the framework and cross-verified provincial assignments against multiple methodological data sources. This process involved systematic cross-verification of our WHO-AIMS-aligned provincial assessments against three complementary evidence streams: (1) the most recent national WHO-AIMS analysis of Pakistan (6); (2) Service Availability and Readiness Assessment (SARA)-based primary care data from Punjab (19); and (3) the EMERALD consortium’s six-country mental health systems assessment, which used a WHO-AIMS-aligned checklist to examine readiness for mental health integration in Ethiopia, India, Nepal, Nigeria, South Africa, and Uganda (20). India was selected as the primary comparator because of shared colonial legislative ancestry and broadly comparable subnational implementation challenges in mental health governance (15, 20). Discrepancies between sources were resolved through discussion and, where necessary, reference to primary WHO documentation. This triangulation approach strengthened confidence in our tier classifications by ensuring provincial assessments were internally consistent across methodologically distinct data sources and externally aligned with patterns observed in structurally similar regional health systems.

### 2.5 Analytical framework: provincial tier classification

To address the decentralized governance landscape following the 18th Constitutional Amendment, we developed a six-criteria provincial readiness framework conceptually anchored in the World Health Organization Assessment Instrument for Mental Health Systems version 2.2 (WHO-AIMS) and informed by the EMERALD consortium situation-analysis checklist used to assess mental health system readiness in six low- and middle-income countries (10, 20). WHO-AIMS comprises six domains, policy and legislative framework, mental health services, mental health in primary care, human resources, public information and links with other sectors, and monitoring and research, operationalized through 28 facets and 155 items (10). Because province-level dementia and mental health data in Pakistan are fragmented and many WHO-AIMS indicators are unavailable at subnational level, we used WHO-AIMS as a conceptual scaffold rather than applying it in full, selecting six proxy criteria judged to be the most reliable indicators of provincial readiness in a data-poor setting and grouped under three dimensions:

1. Governance: Mental Health Authority status (functional, established but non-operational, or not established) and legislative maturity (years since enactment of province- or territory-specific mental health legislation). Both criteria correspond to WHO-AIMS Domain 1, specifically the facets covering mental health authority designation and mental health legislation.
2. Infrastructure: Number of major dedicated psychiatric facilities and geographic distribution of services across the province or territory (multiple cities, single capital, or no systematic distribution). Both criteria correspond to WHO-AIMS Domain 2, specifically the facets covering mental hospitals, community-based psychiatric inpatient units, and equity of geographic access.
3. Specialized care: Presence of dementia-specific services (dedicated dementia day-care, geriatric units, or research centers; embedded general psychiatric or neurological capacity for cognitive-disorder referral; or absent) and diagnostic capacity (multiple operational memory clinics; limited, anecdotal or informal memory clinics; or none). Both criteria correspond to WHO-AIMS Domain 2, specifically the facets covering outpatient facilities, day-treatment facilities, and specialized service availability. The Specialized Care dimension was operationalized on three levels rather than two so that Tier 2 jurisdictions, in which cognitive-disorder care is delivered through general psychiatric or neurological services without dedicated dementia infrastructure, are distinguished from Tier 3 jurisdictions, in which no major dedicated psychiatric institution exists and older adults with cognitive disorders depend on extra-provincial referral. The detailed criterion definitions are provided in Table 1.

**Table 1:** Provincial dementia service categorization framework.

Dimension	Criterion	Tier 1 (established system)	Tier 2 (emerging System)	Tier 3 (absent/nascent system)
Governance	Mental health authority status	Functional: regularly publishes reports, conducts research, and issues communications.	Established (non-operational): authority exists on paper but is inactive or lacks operational output.	Not established: No designated provincial authority exists.
	Legislative maturity	High: legislation enacted at least 10 years ago (Implementation phase).	Medium: legislation enacted at least 5 years ago (Formulation phase).	Minimal/legacy: no provincial/state-specific legislation enacted (may rely on federal ordinance).
Infrastructure	Psychiatric facilities	Multiple: two or more major public psychiatric institutions serving the province.	Single: only one major institution serving the entire provincial population.	None: no major dedicated psychiatric facility.
	Geographic reach	Provincial coverage: services exist in multiple major cities (even if urban-concentrated).	Centralized: services restricted to a single provincial capital city.	No coverage: no systematic service distribution.
Specialized care	Dementia services	Present: dedicated dementia day-care, research center, or geriatric unit exists.	Embedded: no dedicated dementia services; cognitive-disorder referrals are managed within general psychiatric or neurological services at a major tertiary institution.	Absent: no major dedicated psychiatric institution; older adults with cognitive disorders depend on extra-provincial referral.
	Diagnostic capacity	Established: multiple memory clinics operational.	Limited: zero or anecdotal/informal memory clinics only.	None: no memory clinic capacity.

The framework is conceptually anchored in the World Health Organization Assessment Instrument for Mental Health Systems (WHO-AIMS) Domains 1 (policy and legislative framework) and 2 (mental health services); see Methods for the full mapping. All six criteria are equally weighted. The Specialized Care “Embedded” code captures jurisdictions in which cognitive-disorder care is delivered through general psychiatric or neurological services without dedicated dementia infrastructure; the “Absent” code captures jurisdictions with no major dedicated psychiatric institution. Jurisdiction-level application of the framework to Pakistan’s four provinces and two territories is provided in Table 2.

Each province and territory was assessed using the same six criteria, applied systematically to ensure consistency in classification rather than weighted scoring. The selected criteria were Mental Health Authority status, legislative maturity, major psychiatric facility base, geographic distribution of services, presence of dementia-specific services, and diagnostic capacity. Based on performance across these criteria, provinces were stratified into three tiers: (1) Tier 1 (functional governance, multiple facilities, and present specialized services); (2) Tier 2 (non-functional governance, single major facility, centralized service distribution, embedded general psychiatric or neurological capacity for cognitive-disorder referral, and limited or informal diagnostic capacity); and (3) Tier 3 (nascent or absent systems, no Mental Health Authority, no dementia-specific services, and only scattered low-capacity general mental health provision). Two reviewers independently applied the framework to each province and territory; discrepancies in tier assignment were resolved through consensus discussion. These assignments were validated through triangulation with 2024 WHO-AIMS Pakistan data (6).

#### 2.5.1 Rationale for criterion selection

The six criteria were selected on three grounds. First, direct correspondence to the WHO-AIMS domains most relevant to dementia readiness, namely policy and legislative framework (Domain 1) and mental health services (Domain 2). Second, uniform availability of subnational data across all six Pakistani jurisdictions, allowing consistent application. Third, prior precedent in WHO-AIMS-aligned regional health-system situation analyses (2). The remaining WHO-AIMS domains (mental health in primary care; human resources; public information and links with other sectors; monitoring and research) were not directly operationalized as separate criteria because subnational data were unavailable or were addressed indirectly through the workforce density and service-delivery indicators reported in the *Results*.

#### 2.5.2 Equal weighting

All six criteria were treated as equally weighted indicators of provincial readiness. We did not apply a weighted composite score, both because the data-poor setting did not support empirical derivation of weights and because equal weighting is the convention in comparable WHO-AIMS-aligned situation analyses (2). Tier assignments reflect the pattern of performance across the six criteria rather than a numeric aggregate.

#### 2.5.3 Independent rating and consensus procedure

Two authors (SB and SJ) independently applied the framework to each of the six provinces and territories. Each rater assigned a discrete value to each province-by-criterion cell, yielding 36 cells per rater, on the basis of the standardized criterion definitions specified in Table 1. The two raters then compared their assignments cell by cell. Where the two raters disagreed on a cell, the disagreement was discussed with reference to the underlying source documents until consensus was reached. A third author (TQ) reviewed the final tier classifications for clinical and methodological coherence. The final tier assignments were cross-checked through triangulation with the most recent national WHO-AIMS analysis of Pakistan (3), the Service Availability and Readiness Assessment of Punjab primary health care facilities (4), and the EMERALD consortium six-country situation analysis (2). This triangulation is reported in the *Triangulation of Findings with Independent Data Sources* section of the *Discussion*.

#### 2.5.4 Concordance reporting

A formal pre-consensus inter-rater agreement statistic was not calculated. With six provinces and territories and six criteria, the design yields 36 jurisdiction-by-criterion cells, a sample below the threshold at which Cohen’s kappa provides stable estimates with informative confidence intervals: Sim and Wright recommend that reliability studies using kappa for two raters and binary or low-ordinal categorical data report sample sizes substantially greater than 36 to achieve adequate precision, particularly when prevalence of any one category is high (5); McHugh similarly notes that small-sample kappa values are highly sensitive to prevalence imbalance and may produce paradoxically low estimates even with high observed agreement (6); the Landis and Koch interpretive thresholds (7) are most meaningful when applied to samples adequately powered to estimate the underlying agreement. Calculating kappa on 36 cells with the prevalence imbalance typical of LMIC tier classification, in which most Specialized Care items in Tier 2 and Tier 3 jurisdictions code as Embedded or Absent, would therefore be more likely to mislead than to inform. The published precedent for WHO-AIMS-aligned multi-jurisdiction situation analyses is to apply structured consensus and external triangulation rather than formal kappa reporting (2). We followed that precedent and explicitly disclose this design choice in the *Strengths and Limitations* section.

#### 2.5.5 Tier stratification rule

Based on performance across the six criteria, provinces and territories were stratified into three tiers. Tier 1 was defined by a functional Mental Health Authority, mature legislation, multiple major psychiatric facilities, provincial geographic coverage, present specialized dementia services, and established diagnostic capacity. Tier 2 was defined by a non-functional Mental Health Authority, mid-stage legislation, a single major psychiatric facility, centralized services, embedded general psychiatric or neurological capacity capable of receiving cognitive-disorder referrals, and limited or informal diagnostic capacity. Tier 3 was defined by the absence of a designated Mental Health Authority, no province- or territory-specific legislation or only a legacy adaptation of the federal Mental Health Ordinance, no major dedicated psychiatric institution, no systematic service distribution, no dementia-specific services with reliance on extra-provincial referral, and no memory clinic capacity.

## 3 Results

### 3.1 Provincial service categorization using the six-criteria framework

**Figure 1:**
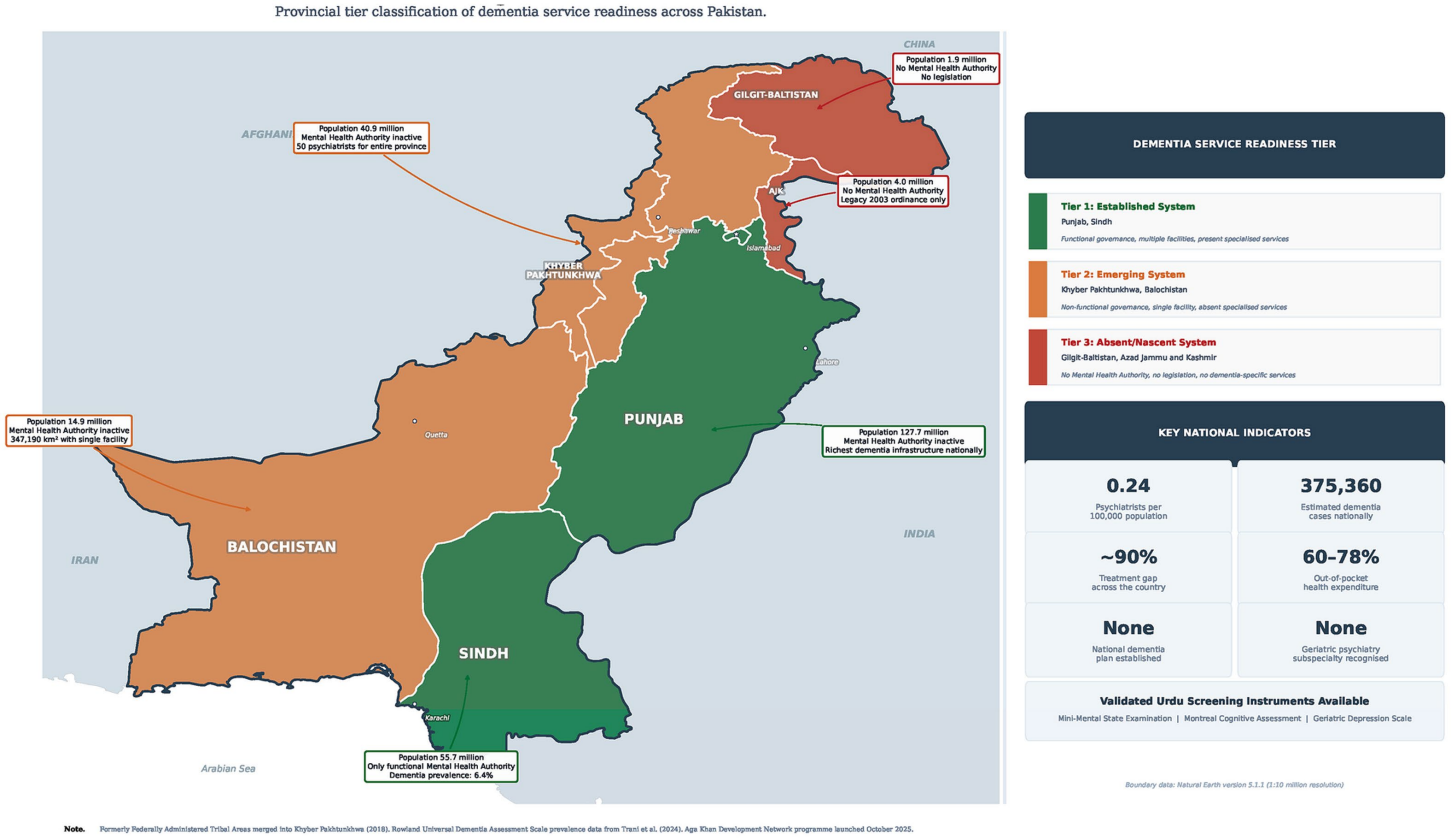
Provincial tier classification of dementia service readiness across Pakistan. Formerly federally administered tribal areas merged into Khyber Pakhtunkhwa (2018). Rowland Universal Dementia Assessment Scale prevalence data from Trani et al. (27). Aga Khan Development Network program launched October 2025. Boundary data from *Natural Earth* version 5.1.1 (1:10 million resolution).

Narrative depth varies partly due to differences in published data across provinces; however, the same six-criteria framework was applied consistently across all territories to ensure comparability in classification (Table 2, Figure 1). To improve transparency, the narrative synthesis below integrates general psychiatric infrastructure and dementia-specific capacity within each province or territory rather than treating them as separate parallel descriptions.

**Table 2:** Provincial assessment of geriatric mental health and dementia services.

Province/tier	Dimension	Status	Key details and gaps
Sindh (tier 1)	Governance	Operational	Mental health authority is functional; publishes reports (2017–21)
	Legislation	Enacted	*Sindh mental health act (2013)*; 11 years of implementation
	Infrastructure	Multi-facility	Includes major institutions such as Karwan-e-Hayat, Karachi Psychiatric Hospital (KPH), and Aga Khan University Hospital; service distribution is geographically concentrated in Karachi
	Dementia care	Available	Geriatric units established; research cohorts present (e.g., 10/66 study)
Punjab (tier 1)	Governance	Established	Authority notified but currently non-operational/inactive
	Legislation	Enacted	*Punjab mental health act (2014)*; 10 years of implementation
	Infrastructure	Multi-facility	Network of 4 major institutes. centralized in Lahore and Rawalpindi
	Dementia care	Available	Documented memory clinic at Mayo hospital, Lahore, plus multiple private clinics advertising memory services in eastern Punjab; Alzheimer’s Pakistan day-care active
Khyber Pakhtunkhwa (tier 2)	Governance	Established	Authority notified but currently non-operational/inactive
	Legislation	Enacted	*KP mental health act (2019)*; 5 years of implementation
	Infrastructure	Single center	Services centralized at Institute of Mental Health Sciences (Peshawar)
	Dementia care	Embedded	No dedicated dementia units; No formal memory clinics
Balochistan (tier 2)	Governance	Established	Authority notified but currently non-operational/inactive
	Legislation	Enacted	*Balochistan mental health act (2019)*; 5 years of implementation
	Infrastructure	Single center	Services centralized at BIPBS (Quetta)
	Dementia care	Embedded	General psychiatry only (limited geriatric expertise available at BIPBS)
Gilgit-Baltistan (tier 3)	Governance	Not established	No mental health authority designated
	Legislation	Not enacted	No legislative framework currently in place
	Infrastructure	Programmatic	AKDN integrated mental health program (launched 2025) plus small general/CMH psychiatry services; no dedicated provincial psychiatric hospital
	Dementia care	None identified	No coverage; reliance on referral to Tier 1 provinces
Azad Jammu and Kashmir (tier 3)	Governance	Not established	No mental health authority designated
	Legislation	Minimal/legacy	Adaptation of federal mental health ordinance 2001 (AJK act 2003); no mental health authority established and little evidence of implementation
	Infrastructure	Limited	No major dedicated psychiatric institution identified
	Dementia care	None identified	No coverage; reliance on referral to Punjab

MHA, mental health authority; KPH, Karachi psychiatric hospital; BIPBS, Balochistan Institute of Psychiatry and Behavioral Sciences; AKDN, Aga Khan development network.

### 3.2 Provincial service categorization: tier 1 provinces

The provinces of Punjab and Sindh demonstrate the highest level of service availability according to the applied framework, although substantial structural deficits persist.

#### 3.2.1 Sindh

Sindh, with a population of 55.7 million, is the only province with a demonstrably functional Mental Health Authority, reflected in progress reports issued between 2017 and 2021 and in the relatively mature implementation environment created by the Sindh Mental Health Act 2013 (6, 21). Service delivery is concentrated in Karachi through Karwan-e-Hayat, Karachi Psychiatric Hospital, Liaquat National Hospital’s senior citizens unit, and Aga Khan University Hospital, which together provide the province’s densest mix of psychiatric, geriatric, and dementia-related services, including outpatient assessment, specialist follow-up, and locally generated dementia research such as tertiary cohorts and Urdu cognitive validation work (22–26). A community-based study sampling adults aged 50 and older across selected rural districts of Punjab and Sindh, using the Rowland Universal Dementia Assessment Scale, reported that 6.4% had moderate-to-severe dementia and a further 20.9% had mild dementia, with significantly higher rates among women (11.4% versus 2.9% in men) and those experiencing multidimensional poverty (27). The authors note that the sample was limited to selected districts of Punjab and Sindh and is not generalizable to Pakistan nationally; these figures should therefore be interpreted as the best available subnational estimate, not as a national prevalence. Despite this relative advantage, access remains heavily metropolitan and does not translate into equitable dementia pathways across rural Sindh.

#### 3.2.2 Punjab

Punjab, home to approximately 127.7 million people, demonstrates the broadest dementia-related infrastructure but not the strongest governance, as its Mental Health Authority remains notified yet functionally inactive despite the Punjab Mental Health Act 2014 (6). The province’s service base is concentrated in Lahore and Rawalpindi and includes major psychiatric institutes, a documented memory clinic at King Edward Medical University, Alzheimer’s Pakistan day-care and outreach services, and a visible private market of neurologists and physicians who currently function as de facto dementia providers for families able to pay (28–30). However, these services remain urban, fragmented, and insufficiently linked to a province-wide referral pathway or long-term care model.

### 3.3 Provincial service categorization: tier 2 provinces

The provinces of Khyber Pakhtunkhwa and Balochistan exhibit moderate capacity, characterized by Mental Health Authorities that are established but inactive, a reliance on single dedicated tertiary psychiatric hospitals, and an absence of specialized dementia services (6).

#### 3.3.1 Khyber Pakhtunkhwa

Khyber Pakhtunkhwa, with a population of approximately 40.86 million, has enacted mental health legislation and constituted a provincial Mental Health Authority, but both governance and specialist service delivery remain weak in practice (6). Care is still centered on Peshawar, particularly the Institute of Mental Health Sciences inaugurated in 2023, with only smaller outpatient psychiatric units elsewhere in general hospitals; no formal memory clinic or dedicated dementia unit was identified, meaning older adults with cognitive decline are likely to rely on general physicians, neurologists, or referral outside routine structured pathways (6, 31).

#### 3.3.2 Balochistan

Balochistan relies overwhelmingly on the Balochistan Institute of Psychiatry and Behavioral Sciences in Quetta, a 130-bedded specialist hospital that functions as the province’s principal psychiatric referral hub, illustrating the extreme centralization of care (6). Although legislation exists, the provincial Mental Health Authority appears inactive and no dedicated dementia clinic, day-care service, or structured memory pathway was identified; consequently, dementia-related care is embedded within general psychiatry and is likely to remain limited in diagnostic depth and continuity, especially outside Quetta (4, 6).

### 3.4 Provincial service categorization: tier 3 territories

The territories of Gilgit-Baltistan and Azad Jammu and Kashmir score lowest on nearly all domains of the assessment framework, reflecting extremely limited governance, workforce, and service capacity. In both settings, dementia readiness is constrained less by isolated clinical gaps than by the near absence of durable mental health architecture, specialist pathways, and province- or territory-level implementation mechanisms.

#### 3.4.1 Gilgit-Baltistan

In Gilgit–Baltistan, mental health governance remains nascent, with no dedicated provincial mental health legislation or Mental Health Authority and historically minimal formal psychiatric infrastructure (32). The recently launched AKDN Integrated Mental Health Program may modestly expand general mental health access, but no dementia-specific service, memory clinic, or routine cognitive assessment pathway was identified; older adults requiring specialist assessment are therefore likely to depend on referral to major centers in Tier 1 provinces (33, 34).

#### 3.4.2 Azad Jammu and Kashmir

Azad Jammu and Kashmir similarly lacks an operational Mental Health Authority and relies on an adapted 2003 version of the federal Mental Health Ordinance 2001 with limited evidence of implementation (35). No major dedicated psychiatric institution or dementia-specific service was identified, and older adults with suspected cognitive disorders appear to depend on fragmented general mental health provision or referral to Punjab, reinforcing the territory’s Tier 3 status for governance, specialist capacity, and dementia care readiness. To our knowledge no peer-reviewed dementia prevalence, incidence, or service-use study has been conducted in Azad Jammu and Kashmir. The 2024 Lancet Commission identifies low educational attainment, social isolation, and limited access to healthcare as among the strongest modifiable risk factors for dementia globally, with the largest population-attributable fractions concentrated in low- and middle-income settings (2); the territory’s combination of nascent governance, absent specialist services, and rural socioeconomic profile is therefore expected to amplify cognitive risk in the absence of direct measurement.

### 3.5 Availability of validated screening instruments

Despite the infrastructural disparities described above, Pakistan possesses culturally adapted screening instruments that are essential for the detection of cognitive decline. The Mini-Mental State Examination (MMSE) has been validated in Urdu by Aga Khan University, with cutoff scores stratified by education level to account for the varying literacy rates across the population (24). Similarly, the Montreal Cognitive Assessment (MoCA) in Urdu has established cutoff scores, where values below 26 indicate mild cognitive impairment and values below 17 indicate dementia (36). The 15-item Geriatric Depression Scale has also been extensively validated in the Urdu language (37). While these diagnostic tools are available for deployment, they have not yet been systematically integrated into routine clinical practice.

### 3.6 Regional comparative analysis

India demonstrates the most developed dementia infrastructure among the comparator nations, although it still lacks a unified national dementia strategy (Table 3). Unlike Pakistan’s devolved health system, India maintains federal coordinating mechanisms through the National Program for the Health Care of the Elderly (NPHCE) and the District Mental Health Program (DMHP), which provide funding streams for geriatric care (38). At the sub-national level, the state of Karnataka declared dementia a public health priority in 2022, launching the Karnataka Brain Health Initiative (KaBHI) to integrate neurological care into primary health centers (39). At the non-governmental level, the Alzheimer’s and Related Disorders Society of India (ARDSI) operates 22 chapters nationwide, providing training and day-care services that supplement state capacity (40).

**Table 3:** South Asian comparative analysis.

Indicator	Pakistan	India	Bangladesh	Sri Lanka
Estimated dementia cases	374,060 (2019 modeled estimate) (54)	~8.8 million (aged 60 and older; 7.4% prevalence, 2018–2020) (78)	~1.4 million (projected to 2025; 8.0% community prevalence aged 60 and older, 2019) (41, 52)	>200,000 cases; ~4% prevalence in semi-urban and suburban populations (43, 44)^†^
National dementia plan	Not established (2)	Not established* (2)	Not established (2)	Not established (2)
Psychiatrists per 100,000 population	0.24 (6)	0.30 (79)	0.07 (16)	0.47 (13)
Coordinating body	None (devolved) (7)	NMHP + NPHCE (federal) (38, 50)	None (16)	National mental health directorate (13)
Memory clinics	Documented memory clinic at Mayo hospital, Lahore; private dementia services offered by neurologists and physicians in Lahore, Karachi, and Islamabad (4, 22, 29, 30, 67, 80)	Multiple (NIMHANS, ARDSI) (40)	Very limited (41)	Limited; concentrated in teaching hospitals (43, 44)
Community workers	Lady health worker program (61, 63)	Accredited social health activists (50)	Community health care providers, staffing community clinics (16)	Public health midwives
NGO presence	Alzheimer’s Pakistan (28)	ARDSI, 22 chapters nationwide (81)	Alzheimer Society of Bangladesh (82)	Sri Lankan Association of Geriatric Medicine and Lanka Alzheimer’s Foundation (43, 83)
Treatment gap	~90% (all mental illness)^‡^ (3, 6)	84.5% (overall mental morbidity) (84)	>80% undetected; largest gap in rural settings (2, 41, 42)	Not reported

Country-level dementia case counts in the first row reflect the most authoritative source available for each setting and are not directly comparable across countries owing to differences in ascertainment method and reference year. Workforce densities for Pakistan and India derive from peer-reviewed national mental health system assessments; figures for Bangladesh and Sri Lanka derive from the 2020 World Health Organization Mental Health Atlas country profiles. ^*^The state of Karnataka launched a Brain Health Initiative in 2022 in the absence of a national dementia plan. ^†^No peer-reviewed national community-based prevalence study of dementia in Sri Lanka has been published to the authors’ knowledge; figures are derived from national-agency statistics. ^‡^The Pakistani treatment-gap figure refers to all mental illness rather than dementia specifically; no published dementia-specific national treatment-gap estimate is available. NIMHANS, National Institute of Mental Health and Neuro Sciences; ARDSI, Alzheimer’s and Related Disorders Society of India.

Bangladesh, despite having a lower GDP per capita than Pakistan, has seen an increasing focus on neurocognitive disorders. Recent national surveys estimate a dementia prevalence of approximately 1.4 million individuals, projected to 2025 on the basis of an 8.0% community prevalence among adults aged 60 and older, with the diagnostic gap remaining profound and over 80% of cases undetected in rural areas (41, 42). While a specific national dementia plan is absent, recent non-communicable disease (NCD) policies have begun to incorporate geriatric mental health components.

Sri Lanka possesses the region’s strongest public health infrastructure, utilizing a centralized National Mental Health Directorate within the Ministry of Health to coordinate services (13). The country maintains a higher psychiatrist-to-population ratio (0.47 per 100,000) compared to its neighbors. However, specialized services remain concentrated; while the National Institute of Mental Health (NIMH) operates a dedicated Psycho-Geriatric Unit, dedicated dementia clinics are largely absent from district-level state hospitals, necessitating reliance on general psychiatric services (43, 44).

## 4 Discussion

The tiered findings reveal a gradient of structural maturity: isolated functionality in Tier 1, partial institutional architecture without operationalization in Tier 2, and absence of governance structures in Tier 3. This gradient reflects not resource scarcity alone but systemic governance fragmentation.

### 4.1 Triangulation of findings with independent data sources

Our provincial tier classifications demonstrated strong convergence with findings from methodologically independent assessments of Pakistan’s mental health system and comparable regional health systems.

#### 4.1.1 Corroboration with WHO-AIMS Pakistan 2024

The most recent national WHO-AIMS analysis independently corroborated our governance domain findings. Consistent with our Tier 1 classification, Sindh’s Mental Health Authority was documented as formally established but only partially functional owing to absent operational rules of business (6). For Tier 2 provinces, the assessment confirmed that Khyber Pakhtunkhwa and Balochistan mental health authorities remain non-functional as of 2023 (6). The workforce density figure employed in our analysis (0.24 psychiatrists per 100,000) derived from their national consultation of 564 psychiatrists, which also documented urban private-sector concentration (6). Provincial facility mapping confirmed Balochistan’s reliance on a single public psychiatric facility (6).

#### 4.1.2 Corroboration with service availability and readiness assessment (SARA) data

Facility-level evidence from Punjab further validated our infrastructure findings (19). The SARA assessment of 25 Basic Health Units revealed that only 32% provide diagnosis or management of non-communicable diseases, 52% experience recurrent electricity and water disruptions, and no cancer services were available (19). These findings corroborate our assessment that despite Punjab’s Tier 1 classification for specialized dementia services, primary care infrastructure remains inadequately equipped for chronic disease management, with service capacity concentrated in urban tertiary centers rather than distributed across the provincial primary care network (19).

#### 4.1.3 Corroboration with EMERALD consortium data

Our regional comparative findings were validated against the EMERALD consortium’s six-country situation analysis, which employed a WHO-AIMS-aligned checklist to assess mental health system readiness in India and five other LMICs (20). The assessment documented India’s federal coordination mechanisms (National Mental Health Program and District Mental Health Program) as enabling factors for primary care integration, a structural advantage absent in Pakistan’s devolved governance model post-18th Amendment (20). Pakistan’s psychiatrist density of 0.24/100,000 remains below India’s 0.30/100,000, with both countries falling dramatically short of the high-income country median of approximately 9 per 100,000 and demonstrating severe urban concentration and inter-provincial disparities (45). The EMERALD findings identified poor governance as a critical barrier to mental health integration across LMICs, with treatment gaps exceeding 75% (45, 46). Countries with functional federal coordination, such as India and South Africa, achieved more consistent implementation of mental health legislation across subnational units, whereas countries with weaker central coordination exhibited the provincial-level variability we document in Pakistan (20, 46).

The convergence of our findings with these methodologically independent assessments, spanning national policy analysis (6), facility-level surveys (19), and multi-country comparative research, strengthens confidence that our tier classifications reflect genuine structural disparities rather than artifacts of selective literature synthesis (20).

### 4.2 Health systems analysis: understanding the policy-implementation gap

Our framework analysis suggests that Pakistan’s dementia service disparities are not merely quantitative but structural, a pattern consistent with broader observations of mental-health-system governance gaps documented in comparable low- and middle-income settings. The application of implementation science frameworks offers one interpretive lens through which the observed gap between policy existence and service delivery can be understood; these interpretations should be read as plausible explanations grounded in the available evidence rather than as causal findings, which would require longitudinal data.

The 18th Constitutional Amendment (2010) devolved health to provincial subjects without commensurate capacity transfer, a pattern the implementation science literature has associated with the emergence of “know-do gaps” (7, 47–49). Unlike India, where the National Mental Health Program (NMHP) provides federal coordination alongside state implementation, Pakistan’s post-devolution architecture has resulted in six provincial systems without a federal coordinating mechanism (50). The contrast is instructive: India’s NMHP enables each of 732 districts to receive allocated mental health budgets through federal-state coordination, whereas Pakistan’s provinces must independently establish, fund, and operationalize mental health services without federal technical support or resource sharing (38).

Our finding that Sindh alone maintains a functional Mental Health Authority, despite four provinces possessing legislative frameworks, is consistent with what implementation science terms the role of “implementation climate,” the organizational readiness and leadership through which policy is operationalized (51). Sindh’s authority has published progress reports, conducted research, and issued communications; conversely, Punjab’s authority exists legally but produces none of these outputs despite similar legislative timelines (6). This variation cannot be explained by legislation alone but requires attention to organizational capacity, political will, and administrative functionality.

### 4.3 Systemic barriers to geriatric care prioritization

Understanding why dementia services remain underdeveloped despite demographic pressures requires an examination of stakeholder interests, resource allocation decisions, and the advocacy landscape.

First, dementia lacks an organized advocacy constituency in Pakistan. Unlike maternal-child health or infectious diseases, no substantial interest group consistently demands geriatric psychiatric services. The *Lancet Commission on Dementia* notes that in many Low- and Middle-Income Countries (LMICs), mental health is frequently de-prioritized in healthcare planning, with only 23% of countries investing resources in monitoring mental health initiatives (52, 53). Pakistan exemplifies this pattern; despite an estimated 374,060 dementia cases, Alzheimer’s Pakistan remains the sole national advocacy organization, operating with limited reach (28, 54).

Second, competing health priorities in resource-constrained settings systematically disadvantage non-communicable diseases affecting older adult(s) populations. Pakistan’s total health expenditure represents only 2.8–2.95% of GDP, with out-of-pocket expenditure constituting approximately 60–78% of total health costs (55). In this context, policymakers face difficult allocation trade-offs between maternal mortality reduction, infectious disease control, and geriatric services.

Third, workforce deficits are compounded by the migration of skilled professionals. With geriatric psychiatry yet to be recognized as a distinct sub-specialty by the College of Physicians and Surgeons Pakistan, the primary pathway to specialized training is international fellowship (56). Physicians who obtain such training often encounter strong incentives to practice abroad, contributing to the country’s critically low specialist density (57). Our findings indicate a psychiatric workforce density (0.24 per 100,000) insufficient for projected dementia burden (6). However, we did not conduct primary workforce assessment; these figures derive from published WHO-AIMS analyses and recent national consultations. Validation through dedicated workforce mapping studies is warranted.

### 4.4 Multi-channel service delivery model

**Figure 2:**
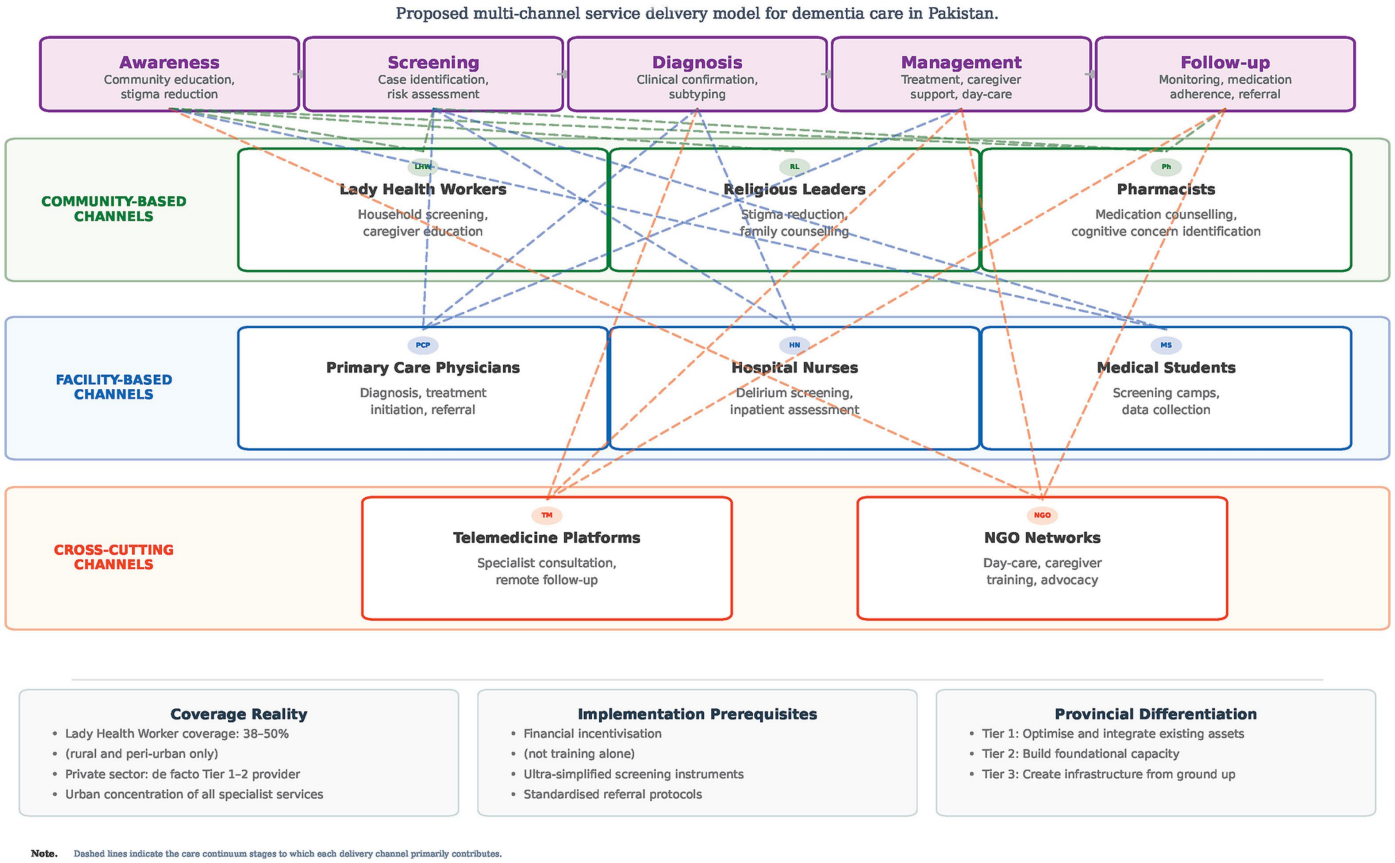
Proposed multi-channel service delivery model for dementia care in Pakistan. Dashed lines indicate the care continuum stages to which each delivery channel primarily contributes. LHW, lady health worker; ID, identification.

Given Pakistan’s severe specialist shortage and fragmented health system, dementia care implementation cannot rely on any single delivery mechanism. We propose a multi-channel service delivery model that leverages diverse existing infrastructure while building new capacity (Figure 2). This approach recognizes that different channels serve complementary functions across the care continuum from awareness to diagnosis to ongoing management (53). This model has not been empirically tested in the Pakistani context; rather, it aligns proposed interventions with existing structures (Lady Health Workers, primary care physicians, telemedicine platforms, pharmacists) identified through our review. Future implementation research is needed to evaluate the effectiveness and feasibility of these proposed channels across different provincial contexts.

### 4.5 Channel-specific implementation strategies

#### 4.5.1 Primary care physicians

Pakistan’s approximately 299,113 registered doctors represent the backbone of diagnostic capacity (58). Integration of dementia recognition into primary care requires: (a) incorporation of the WHO mhGAP dementia module into continuing medical education; (b) provision of validated Urdu screening instruments (MMSE-Urdu, MoCA-Urdu) with interpretation guidance (24, 36); (c) clear referral pathways to neurologists or psychiatrists; and (d) inclusion of cholinesterase inhibitors in essential medicines lists at the primary care level (59). The state infrastructure, comprising 5,527 Basic Health Units (BHUs) and 686 Rural Health Centers (RHCs), provides a potential delivery network, although current utilization for mental health services remains minimal (60).

#### 4.5.2 Community health workers (lady health workers)

The Lady Health Worker Program, established in 1994 and currently employing over 85,000 workers, represents a significant, though not comprehensive, infrastructure (61). Each LHW covers approximately 1,000 people through weekly household visits. The mPareshan project in Badin District has demonstrated the feasibility of mobile app-based mental health interventions delivered by LHWs (62). However, Lady Health Workers already carry substantial maternal-child health and broader public-health responsibilities, making task-shifting fatigue a realistic implementation risk. Evaluation evidence suggests that expanding their remit can compromise core duties, so extending dementia screening to this workforce would require ultra-brief tools, protected supervision, and likely financial incentivization rather than additional training alone. Their coverage reaches around 38% of the population in current strategic planning (primarily in rural and urban-informal settlement settings), necessitating alternative channels for urban and otherwise uncovered populations (63, 64).

#### 4.5.3 Pharmacists

Pakistan’s pharmacy network extends across urban and rural areas, with pharmacists maintaining regular contact with older adult(s) patients obtaining chronic disease medications. Studies in comparable LMIC settings suggest community pharmacists can effectively recognize cognitive warning signs during medication consultations and provide adherence counseling (65). While currently underutilized in this role, specific training modules could enable pharmacists to identify drug interactions relevant to cognitive function and facilitate referrals to primary care physicians.

#### 4.5.4 Telemedicine

Digital health platforms offer a mechanism to bypass geographic barriers, connecting patients in Tier 2 and 3 provinces to specialists concentrated in Karachi and Lahore. The rapid growth of platforms such as *Sehat Kahani* demonstrates the viability of tele-health in the Pakistani context (66). Telemedicine is particularly valuable for specialist consultation when local expertise is unavailable and for the supervision of community health workers in remote areas.

#### 4.5.5 NGO networks

Existing organizations including Alzheimer’s Pakistan, Karwan-e-Hayat, and ConsidraCare Pakistan (private home-based senior and dementia care services) provide specialized expertise absent in government services (28, 67, 68). These entities are best positioned to deliver caregiver training programs, day-care services, and advocacy for policy change, though their sustainability relies on public-private partnership models.

#### 4.5.6 Religious leaders

Given the significant role of religious authority in Pakistani society and documented stigma associating mental illness with spiritual causes (e.g., *Jinn* or black magic), the engagement of religious leaders could substantially impact help-seeking behavior (69, 70). Imams and religious scholars could communicate that dementia is a medical condition rather than a spiritual failing, clarify religious exemptions for patients unable to fulfill obligations, and encourage families to seek medical care.

### 4.6 Tier-specific implementation roadmap

Addressing the structural inequities identified in this review requires a stratified approach to policy implementation that explicitly combines public, quasi-public, and private care pathways. A monolithic national strategy is unlikely to succeed given the heterogeneity of health system maturity across provinces and the reality that dementia care is already accessed through mixed financing and provider arrangements.

A further implication of these findings is that dementia care in Pakistan is already partly privatized. In Tier 1 provinces and parts of Tier 2, private neurologists, psychiatrists, and general physicians often serve as the first point of assessment for families able to pay, particularly where public memory services are absent, fragmented, or poorly linked to referral systems (4, 9). Any implementation roadmap must therefore engage the private sector through referral standards, shared screening pathways, and incentives for continuity and reporting, rather than assuming that public infrastructure alone will absorb demand.

**Figure 3:**
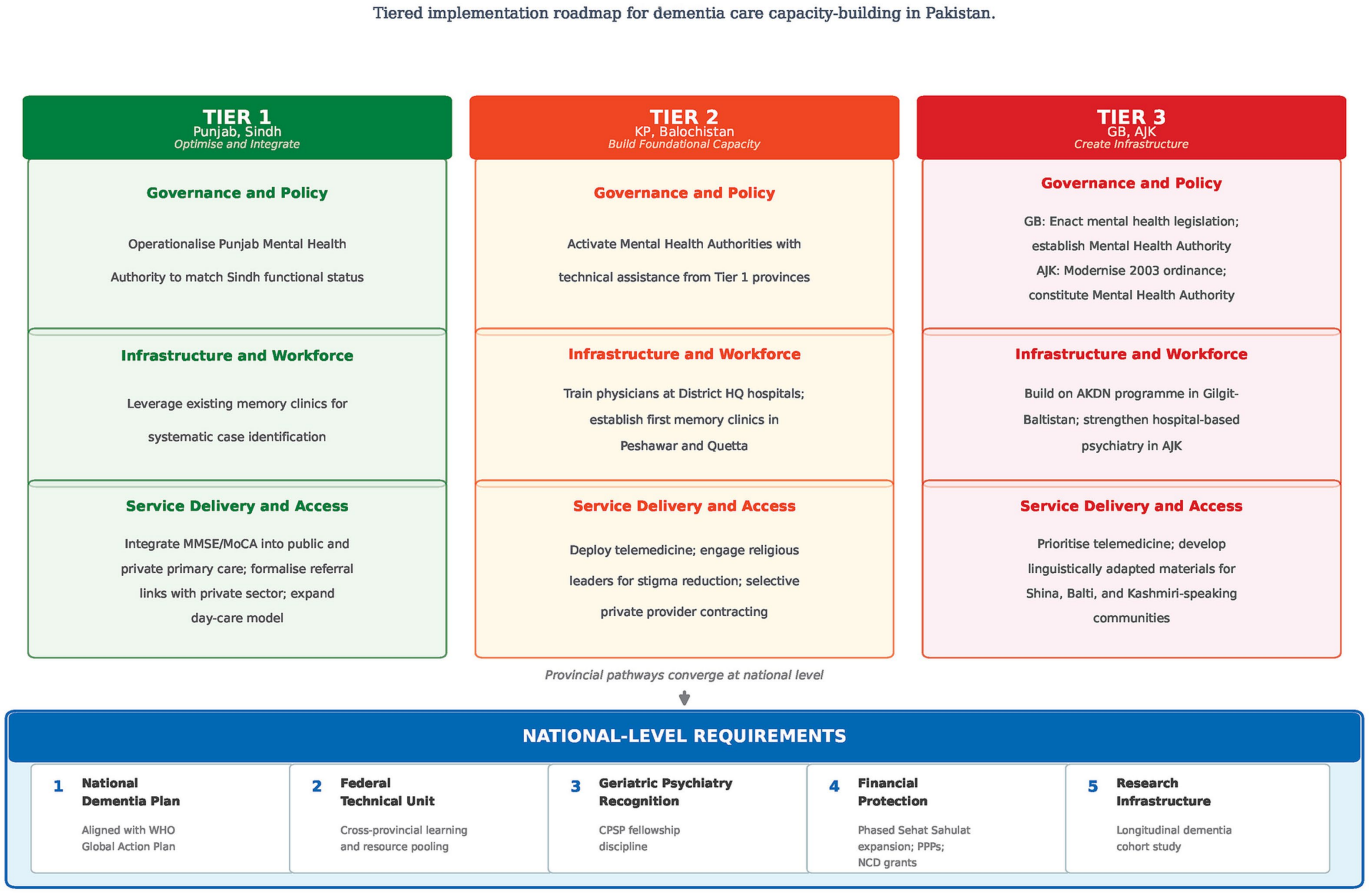
Tiered implementation roadmap for dementia care capacity-building in Pakistan. MHA, mental health authority; KP, Khyber Pakhtunkhwa; GB, Gilgit-Baltistan; AJK, Azad Jammu and Kashmir; AKDN, Aga Khan development network; CPSP, college of physicians and surgeons Pakistan; PPP, public-private partnership; NCD, non-communicable disease.

As detailed in Table 4 and Figure 3, recommendations are graduated: Tier 1 provinces (Punjab, Sindh) can focus on optimization and integration by linking existing public services, NGOs, and private neurologists or general physicians into a more coherent referral network. Tier 2 provinces (Khyber Pakhtunkhwa, Balochistan) require fundamental capacity building, including sentinel public services in provincial capitals supported by telemedicine and selective engagement of private providers. Tier 3 territories (Gilgit-Baltistan, Azad Jammu and Kashmir), facing the most acute deficits, require infrastructure creation and remote-care models capable of overcoming geography and workforce scarcity.

**Table 4:** Tier-specific implementation recommendations.

Target region	Governance and policy	Infrastructure and workforce	Service delivery and access
Tier 1 *(Punjab, Sindh)*	Operationalize Punjab’s MHA to match Sindh’s functional status (7)	Leverage existing memory clinics for systematic case identification	Integrate MMSE/MoCA into public and private primary care; formalize referral links with private neurologists and physicians; expand Alzheimer’s Pakistan day-care model
Tier 2 *(KP, Balochistan)*	Activate MHAs with technical assistance from Tier 1 provinces	Train physicians at District HQ hospitals; establish first memory clinics in Peshawar/Quetta	Deploy telemedicine for remote access; engage religious leaders for stigma reduction; use selective contracting or referral networks with private providers in provincial capitals
Tier 3 *(Gilgit-Baltistan, Azad Jammu and Kashmir)*	• Gilgit–Baltistan: Enact dedicated mental health legislation and establish a Mental Health Authority (32)	Build on the 2025 AKDN Integrated Mental Health Program in Gilgit-Baltistan; strengthen small hospital-based psychiatry units in AJK	Prioritize telemedicine; develop linguistically adapted materials for Shina, Balti, and Kashmiri-speaking communities
	• Azad Jammu and Kashmir: Operationalize and modernize the 2003 adaptation of the Mental Health Ordinance; constitute the Mental Health Authority required under section 3 (1) (85)		
National level	Adopt national dementia plan by 2026-2027; establish federal technical unit	Secure CPSP recognition for geriatric psychiatry fellowship (56)	Pursue phased dementia coverage through Sehat Sahulat or successor schemes, complemented by public-private partnerships and external NCD financing where needed

MHA, mental health authority; KP, Khyber Pakhtunkhwa; GB, Gilgit-Baltistan; AKDN, Aga Khan development network; CPSP, College of Physicians and Surgeons Pakistan.

### 4.7 National-level requirements

While provincial autonomy is central to Pakistan’s post-18th Amendment governance structure, provincial actions must be underpinned by federal coherence, realistic financing, and a mixed-provider implementation logic. Beyond the tier-specific actions outlined above, five national-level measures are essential:

1. Policy adoption: The formulation of a National Dementia Plan by mid-2026, aligned with the targets of the WHO *Global Action Plan on the Public Health Response to Dementia 2017–2025* (71).
2. Coordination: The establishment of a Federal Technical Unit to facilitate cross-provincial learning, standardize training curricula for community health workers, and manage resource pooling.
3. Specialty recognition: The formal recognition of geriatric psychiatry as a distinct fellowship discipline by the College of Physicians and Surgeons Pakistan (CPSP) (56).
4. Financial protection: Where fiscally feasible, dementia diagnostics and selected essential medicines should be incorporated into Sehat Sahulat or successor provincial risk-protection schemes. Given recent financing instability and interruptions affecting insurance coverage, implementation may need phased provincial pilots, public-private partnerships, and supplementary non-communicable disease or donor financing rather than assuming immediate nationwide expansion (35, 72, 73).
5. Research infrastructure: The launch of Pakistan’s first longitudinal dementia cohort study to generate indigenous epidemiological data.

### 4.8 Strengths and limitations

This review has several important strengths. It addresses a major evidence gap by providing, to our knowledge, the first structured synthesis of dementia-relevant geriatric mental health infrastructure across Pakistan at the provincial and territorial level. By integrating peer-reviewed literature with policy documents, governmental reports, and other grey literature, the review captures dimensions of service organization, governance, and implementation that are often missed in conventional academic reviews alone. A further strength is the use of a transparent six-criteria framework, informed by WHO-AIMS conceptual domains, to enable systematic comparison across jurisdictions and to translate findings into a practical tiered implementation model. The inclusion of regional comparisons also strengthens the manuscript by situating Pakistan’s service landscape within a broader health-systems context.

This review has several limitations. First, the evidence base for Pakistani dementia research is thin, with no clinical trials, limited epidemiological studies, and heavy reliance on grey literature. Second, our six-criteria framework, while informed by WHO-AIMS conceptual domains, represents a novel application that has not been formally validated and requires prospective assessment. We also did not calculate a formal pre-consensus inter-rater agreement statistic; the design yields 36 jurisdiction-by-criterion cells, a sample below the threshold at which Cohen’s kappa provides stable estimates, and we therefore followed the precedent of comparable WHO-AIMS-aligned multi-jurisdiction situation analyses in applying structured consensus and external triangulation rather than formal kappa reporting (20, 74, 75). Prospective application of the framework in a larger comparator set would permit formal psychometric evaluation. Third, Tier 3 territory data was particularly limited, potentially underestimating or overestimating actual service availability.

Fourth, the regional comparator countries (India, Bangladesh, Sri Lanka) differ from Pakistan in health-system financing, data infrastructure, case-ascertainment methods, and governance architecture, and their respective dementia evidence bases also vary considerably in maturity and source type (17, 76, 77). India draws on national policy programs (NMHP, NPHCE) and a comparatively developed peer-reviewed evidence base; Bangladesh has recent peer-reviewed national prevalence data (42) but limited service-mapping evidence; Sri Lanka relies primarily on national statistics from the National Institute of Mental Health and the country Alzheimer society (43, 44), with no peer-reviewed national community-prevalence study to our knowledge. Country-level case counts in Table 3 are therefore triangulated against modeled estimates from the Global Burden of Disease 2019 dementia forecast (54). The cross-country comparison in this review should be read as contextual scaffolding for situating Pakistan’s structural position, not as an equivalence test between health systems.

Fifth, we did not conduct primary data collection, relying instead on the synthesis of existing sources. Sixth, the proposed multi-channel delivery model requires empirical testing to determine optimal channel combinations across different provincial contexts. Finally, the review is limited by linguistic bias, as available health system documentation is predominantly in English and Urdu. The absence of materials in regional languages may underrepresent informal or community-level dementia initiatives, particularly in Khyber Pakhtunkhwa, Balochistan, Gilgit-Baltistan, and Azad Jammu and Kashmir, where health documentation practices differ substantially from those in the major provinces.

## 5 Conclusion

Pakistan faces an imminent dementia care crisis that will intensify without urgent intervention. With 374,060 estimated cases and projections indicating the older adult(s) population will triple to 38.3 million by 2050, the nation possesses a narrow demographic window to establish necessary infrastructure before demand overwhelms capacity. Our six-criteria analytical framework, applied for the first time to our knowledge at the provincial level, reveals that current service disparities are not merely resource-based but reflect fundamental health systems governance failures following the devolution under the 18th Constitutional Amendment.

Regional benchmarking demonstrates that Pakistan’s fragmented provincial approach leaves it structurally disadvantaged compared to India’s federally coordinated *National Program for Health Care of the Elderly* and Sri Lanka’s centralized *National Mental Health Directorate*. Yet, Pakistan possesses diverse, underutilized assets. We propose a multi-channel service delivery model that leverages primary care physicians for diagnosis, community health workers for screening, pharmacists for medication adherence, and religious leaders for stigma reduction to bridge the severe specialist gap.

Bridging the identified “know-do gap” requires shifting from siloed provincial efforts to a coordinated national response. Immediate priorities must include the recognition of geriatric psychiatry as a formal sub-specialty, the operationalization of all provincial Mental Health Authorities, and the adoption of a National Dementia Plan to enable cross-provincial resource sharing. A coordinated, multi-channel approach offers Pakistan a feasible path to building dementia care capacity, before demographic pressures become unmanageable, allowing the country to meet the needs of an aging, diverse population across the six territories with fundamentally different implementation requirements.
