# Supplementary material for: Dementia service readiness in Pakistan: a provincial health systems assessment using a novel WHO-AIMS aligned framework
Source: Front Public Health. 2026 Jun 29;14:1837649. doi: 10.3389/fpubh.2026.1837649 (PMC13361205; doi:10.3389/fpubh.2026.1837649)
Supplement: Supplementary file 1 [file Table_1.DOCX]

Supplementary Material

**Supplementary Table S1.** Characteristics and Appraisal of Included Sources.

| **Ref.** | **Source** | **Source Type** | **Geography** | **Year** | **Domain(s)** | **Framework Relevance** | **Source Appraisal** |
| --- | --- | --- | --- | --- | --- | --- | --- |
| 1 | UN DESA, 2024 | International agency report | Global/Pakistan | 2024 | Epidemiology | Demographic projections | High: Official UN statistical publication; standardised methodology |
| 2 | Livingston et al., 2024 | Peer-reviewed article | Global | 2024 | Epidemiology; Policy | Lancet Commission recommendations | High: Peer-reviewed; Lancet Commission; high citation impact |
| 3 | Wang, 2025 | Peer-reviewed article | Global | 2025 | Epidemiology | GBD 2021 dementia burden | High: Peer-reviewed; GBD consortium data |
| 4 | Ali et al., 2023 | Peer-reviewed article | National (Pakistan) | 2023 | Epidemiology; Policy | Pakistan dementia overview | High: Peer-reviewed; Frontiers in Dementia |
| 5 | Khan Q, 2014 | Peer-reviewed article | National (Pakistan) | 2014 | Epidemiology | Dementia practice challenges | High: Peer-reviewed; Neurology (AAN) |
| 6 | Dayani et al., 2024 | Peer-reviewed article | National (Pakistan) | 2024 | Governance; Infrastructure; Workforce | WHO-AIMS Pakistan; MHA status; workforce; triangulation source | High: Peer-reviewed; WHO-AIMS methodology; most current national assessment |
| 7 | Nishtar et al., 2013 | Peer-reviewed article | National (Pakistan) | 2013 | Governance; Policy | 18th Amendment health devolution | High: Peer-reviewed; The Lancet |
| 8 | RSIL, 2020 | Grey literature (policy report) | National (Pakistan) | 2020 | Governance | Inter-provincial coordination | Moderate: Established legal research body; not peer-reviewed; policy-focused |
| 9 | Khan O et al., 2025 | Book chapter | National (Pakistan) | 2025 | Specialised care; Workforce | Geriatric psychiatry in Pakistan | High: Peer-reviewed book chapter; Springer Nature |
| 10 | Saxena et al., 2007 | Peer-reviewed article | Global | 2007 | Governance; Infrastructure | WHO-AIMS methodology | High: Peer-reviewed; WHO-authored; foundational methodology paper |
| 11 | Lora et al., 2016 | Peer-reviewed article | Global | 2016 | Governance | Mental health information systems | High: Peer-reviewed; Epidemiology and Psychiatric Sciences |
| 12 | WHO MH Atlas: Sri Lanka, 2021 | International agency report | Sri Lanka | 2021 | Governance; Infrastructure; Workforce | Regional comparator | High: Official WHO publication; standardised methodology |
| 13 | WHO MH Atlas: Pakistan, 2021 | International agency report | National (Pakistan) | 2021 | Governance; Infrastructure; Workforce | National baseline | High: Official WHO publication; standardised methodology |
| 14 | Younas & Qureshi, 2025 | Peer-reviewed article | Pakistan/India | 2025 | Governance | Colonial legislative comparison | High: Peer-reviewed; World Medical and Health Policy |
| 15 | WHO MH Atlas: Bangladesh, 2021 | International agency report | Bangladesh | 2021 | Governance; Infrastructure; Workforce | Regional comparator | High: Official WHO publication; standardised methodology |
| 16 | Farina et al., 2020 | Peer-reviewed article | Multi-country | 2020 | Epidemiology | STRiDE dementia prevalence | High: Peer-reviewed; systematic review and meta-analysis |
| 17 | WHO GDO, 2018 | International agency report | Global | 2018 | Epidemiology; Policy | Global Dementia Observatory | High: Official WHO publication |
| 18 | Mugisha et al., 2017 | Peer-reviewed article | Multi-country (6 LMICs) | 2017 | Governance; Infrastructure | EMERALD situation analysis; triangulation source | High: Peer-reviewed; WHO-AIMS-aligned methodology |
| 19 | Sindh Mental Health Ordinance, 2013 | Legislation | Provincial (Sindh) | 2013 | Governance | Sindh MHA legislative basis | High: Official provincial legislation |
| 20 | AKU Hospital, 2024 | Institutional website | Provincial (Sindh) | 2024 | Specialised care | Karachi psychiatric services | Moderate: Official institutional website; not peer-reviewed; direct provider |
| 21 | Aoki et al., 2022 | Peer-reviewed article | Global | 2022 | Specialised care | Shared decision-making | High: Peer-reviewed; Cochrane systematic review |
| 22 | Awan et al., 2015 | Peer-reviewed article | National (Pakistan) | 2015 | Specialised care | MMSE-Urdu validation | High: Peer-reviewed; validation study |
| 23 | Khan QUA et al., 2020 | Peer-reviewed article | Provincial (Sindh) | 2020 | Specialised care | 10/66 Urdu validation | High: Peer-reviewed; Alzheimer Disease and Associated Disorders |
| 24 | Liaquat National Hospital, 2024 | Institutional website | Provincial (Sindh) | 2024 | Specialised care | Senior citizens unit | Moderate: Official hospital website; not peer-reviewed; direct provider |
| 25 | Trani et al., 2024 | Peer-reviewed article | Multi-country incl. Pakistan | 2024 | Epidemiology | Poverty and dementia association | High: Peer-reviewed; Innovation in Aging |
| 26 | ADI: Alzheimer’s Pakistan, n.d. | NGO website | National (Pakistan) | n.d. | Specialised care; Policy | Alzheimer’s Pakistan profile | Moderate: Established international NGO (ADI); not peer-reviewed; advocacy source |
| 27 | KEMU, 2024 | Institutional website | Provincial (Punjab) | 2024 | Specialised care | Psychiatry department | Moderate: Official university hospital website; not peer-reviewed |
| 28 | SehatYab, 2026 | Institutional website | National (Pakistan) | 2026 | Specialised care | Private elderly care | Low: Commercial healthcare platform; not peer-reviewed; self-reported data |
| 29 | Dawn, 2023 | News article | Provincial (KP) | 2023 | Infrastructure | KP mental health institute | Low: News media; factual reporting of inauguration event |
| 30 | Daily Independent, 2025 | News article | Gilgit-Baltistan | 2025 | Infrastructure | AKDN programme launch | Low: News media; factual reporting of programme launch |
| 31 | The Nation, 2025 | News article | Gilgit-Baltistan | 2025 | Infrastructure | AKDN programme launch | Low: News media; corroborates [30]; factual reporting |
| 32 | Hafeez et al., 2023 | Peer-reviewed article | National (Pakistan) | 2023 | Epidemiology; Infrastructure | GBD 2019 Pakistan | High: Peer-reviewed; Lancet Global Health; GBD data |
| 33 | Fahd et al., 2020 | Peer-reviewed article | National (Pakistan) | 2020 | Specialised care | GDS-Urdu validation | High: Peer-reviewed; validation study |
| 34 | MoHFW India, 2010 | Government report | India | 2010 | Governance; Infrastructure | NPHCE programme | High: Official government programme documentation |
| 35 | Mailankody et al., 2024 | Peer-reviewed article | India (Karnataka) | 2024 | Infrastructure; Specialised care | KaBHI training | High: Peer-reviewed; JFMPC |
| 36 | ARDSI, 2020 | NGO report | India | 2020 | Specialised care; Policy | Dementia in India | Moderate: Established national NGO; systematic data compilation |
| 37 | Naheed et al., 2023 | Peer-reviewed article | Bangladesh | 2023 | Epidemiology | Bangladesh dementia prevalence | High: Peer-reviewed; Lancet Regional Health |
| 38 | Global Health Network Asia, 2021 | Grey literature | Bangladesh | 2021 | Epidemiology | Bangladesh rural dementia | Moderate: Research network; workshop proceedings; limited detail |
| 39 | NIMH Sri Lanka, n.d. | Institutional website | Sri Lanka | n.d. | Epidemiology | Sri Lanka dementia statistics | Moderate: Official national mental health institute; undated |
| 40 | NIMH Sri Lanka, 2013 | Institutional report | Sri Lanka | 2013 | Infrastructure | NIMH annual report | Moderate: Official national institute; dated; limited to single year |
| 41 | Rashid et al., 2023 | Peer-reviewed article | Provincial (Punjab) | 2023 | Infrastructure | SARA BHU assessment; triangulation source | High: Peer-reviewed; Frontiers in Public Health; facility-level data |
| 42 | WHO MH Atlas, 2025 | International agency report | Global | 2025 | Governance; Workforce | Mental Health Atlas 2024 | High: Official WHO publication; most current global data |
| 43 | Semrau et al., 2019 | Peer-reviewed article | Multi-country | 2019 | Governance; Policy | EMERALD recommendations | High: Peer-reviewed; BJPsych Open |
| 44 | Proctor et al., 2009 | Peer-reviewed article | Global | 2009 | Policy | Implementation science | High: Peer-reviewed; foundational implementation science paper |
| 45 | Ahmed et al., 2022 | Peer-reviewed article | India | 2022 | Governance; Infrastructure | India NMHP systematic review | High: Peer-reviewed; systematic review |
| 46 | Aarons et al., 2012 | Peer-reviewed article | United States | 2012 | Policy | Implementation climate | High: Peer-reviewed; Implementation Science |
| 47 | Livingston et al., 2020 | Peer-reviewed article | Global | 2020 | Policy | Lancet Dementia Commission 2020 | High: Peer-reviewed; The Lancet |
| 48 | WHO, 2022 | International agency report | Global | 2022 | Policy | World Mental Health Report | High: Official WHO flagship publication |
| 49 | Nichols et al., 2022 | Peer-reviewed article | Global | 2022 | Epidemiology | GBD 2019 dementia projections | High: Peer-reviewed; Lancet Public Health; GBD data |
| 50 | CPSP, 2024 | Institutional website | National (Pakistan) | 2024 | Workforce; Specialised care | Fellowship programmes | Moderate: Official regulatory body; not peer-reviewed; authoritative for training data |
| 51 | Tahir et al., 2011 | Peer-reviewed article | National (Pakistan) | 2011 | Workforce | Brain drain of doctors | High: Peer-reviewed |
| 52 | GoP Finance Division, 2024 | Government report | National (Pakistan) | 2024 | Infrastructure; Workforce | Pakistan Economic Survey 2023–24 | High: Official government statistical publication |
| 53 | MoCA Cognition, 2015 | Institutional website | Global | 2015 | Specialised care | MoCA-Urdu test form | Moderate: Official MoCA website; standardised instrument source |
| 54 | WHO, 2016 | International agency report | Global | 2016 | Specialised care; Infrastructure | mhGAP intervention guide | High: Official WHO clinical guideline |
| 55 | GoP Finance Division, 2019 | Government report | National (Pakistan) | 2019 | Infrastructure | Pakistan Economic Survey 2018–19 | High: Official government statistical publication |
| 56 | Hafeez et al., 2011 | Peer-reviewed article | National (Pakistan) | 2011 | Infrastructure; Workforce | LHW programme review | High: Peer-reviewed; JPMA |
| 57 | Rabbani et al., 2024 | Peer-reviewed article | Provincial (Sindh) | 2024 | Infrastructure; Specialised care | mPareshan LHW trial | High: Peer-reviewed; JMIR Research Protocols |
| 58 | GoP, 2022 | Government report | National (Pakistan) | 2022 | Infrastructure; Workforce | LHW Strategic Plan 2022–2028 | High: Official government strategic plan |
| 59 | Oxford Policy Management, 2019 | Grey literature (evaluation) | National (Pakistan) | 2019 | Infrastructure | LHW performance evaluation | Moderate: Established international consultancy; commissioned evaluation |
| 60 | Azhar et al., 2009 | Peer-reviewed article | National (Pakistan) | 2009 | Workforce | Pharmacist role | High: Peer-reviewed; Human Resources for Health |
| 61 | Saifullah et al., 2024 | Peer-reviewed article | National (Pakistan) | 2024 | Infrastructure | Telehealth services | High: Peer-reviewed; Frontiers in Public Health |
| 62 | ConsidraCare, 2026 | Institutional website | National (Pakistan) | 2026 | Specialised care | Private dementia care | Low: Commercial provider website; self-reported services |
| 63 | Karwan-e-Hayat, 2024 | Institutional website | Provincial (Sindh) | 2024 | Specialised care | Day care services | Moderate: Established NGO hospital; not peer-reviewed; direct provider |
| 64 | Daraz et al., 2025 | Peer-reviewed article | Provincial (KP) | 2025 | Policy | Mental health stigma | High: Peer-reviewed; Frontiers in Psychiatry |
| 65 | Nisar et al., 2019 | Peer-reviewed article | Provincial (Sindh) | 2019 | Policy | Perceptions of depression | High: Peer-reviewed; Cureus |
| 66 | WHO, 2017 | International agency report | Global | 2017 | Policy | Global Action Plan on Dementia | High: Official WHO policy document |
| 67 | Kiani, 2026a | News article | National (Pakistan/AJK/GB) | 2026 | Policy; Infrastructure | Health coverage for AJK, GB | Low: News media; factual reporting of policy development |
| 68 | Kiani, 2026b | News article | National (Pakistan) | 2026 | Policy | Federal health scheme funding | Low: News media; factual reporting of financing decision |
| 69 | Ajmal et al., 2024 | Peer-reviewed article | National (Pakistan) | 2024 | Governance | Mental health law analysis | High: Peer-reviewed; Pakistan Social Sciences Review |
| 70 | AJK Legislative Assembly, 2003 | Legislation | AJK | 2003 | Governance | AJK Mental Health Ordinance | High: Official legislation |
| 71 | Khattak et al., 2024 | Peer-reviewed article | Provincial (Punjab) | 2024 | Epidemiology; Specialised care | MoCA-Urdu cutoff scores; cognitive impairment prevalence in Rawalpindi | High: Peer-reviewed; Journal of Bahria University Medical and Dental College |
| 72 | World Bank, 2024 | International agency report | National (Pakistan) | 2024 | Policy; Infrastructure | Pakistan health expenditure as percentage of GDP | High: Official World Bank open data indicator; standardised cross-country methodology |

**Note.** Supplementary Table S1 catalogues the 72 sources included in the integrative evidence synthesis. Additional references in the manuscript support methodology, reporting, or interpretation and are therefore not all included in this source-appraisal table. Source appraisal ratings reflect the authority and reliability of each source using a simplified framework adapted for integrative reviews incorporating grey literature. **High**: peer-reviewed indexed journal, official WHO/UN publication, official government publication, or enacted legislation. **Moderate**: established institutional or organisational source with recognised authority (e.g., official hospital website, regulatory body, established NGO, commissioned evaluation) but not peer-reviewed. **Low**: news media or commercial sources used solely for factual reporting of events, programme launches, or service descriptions; findings from these sources were corroborated against higher-authority sources where possible. n.d. = no date.

**Supplementary Table S2.** Search Strategy, Data Sources, Eligibility Criteria, and Screening Summary.

**Panel A. Database and Supplementary Search Strategy.**

| **Database** | **Search String** | **Date Restrictions** | **Last Search Date** | **Results Retrieved** |
| --- | --- | --- | --- | --- |
| PubMed | ("dementia" OR "Alzheimer" OR "cognitive impairment" OR "geriatric psychiatry" OR "memory clinic") AND ("Pakistan" OR "Punjab" OR "Sindh" OR "Khyber Pakhtunkhwa" OR "Balochistan" OR "Gilgit-Baltistan" OR "Azad Jammu and Kashmir") AND ("mental health" OR "health system" OR "legislation" OR "workforce" OR "screening") | None | 9 Nov 2025; updated 16 Mar 2026 | 263 |
| Scopus | TITLE-ABS-KEY(("dementia" OR "Alzheimer*" OR "cognitive impairment" OR "geriatric psychiatry" OR "memory clinic") AND ("Pakistan" OR "Punjab" OR "Sindh" OR "Khyber Pakhtunkhwa" OR "Balochistan" OR "Gilgit-Baltistan" OR "Azad Kashmir") AND ("mental health" OR "health system*" OR "legislation" OR "workforce" OR "screening")) | None | 9 Nov 2025; updated 16 Mar 2026 | 65 |
| Google Scholar | Multiple iterative searches using combinations of the following terms in the advanced search interface: "dementia", "Alzheimer", "cognitive impairment", "geriatric psychiatry", "memory clinic" combined with "Pakistan", "mental health", "health system", "screening", "legislation", "workforce", and individual province or territory names. Seven query combinations were executed. The first 20 pages of results were screened per query combination. | None | 10 Nov 2025; updated 16 Mar 2026 | First 20 pages per query (7 queries) |
| Supplementary searches | Province-specific supplementary searches using combinations of individual province or territory names with: "mental health authority", "psychiatric hospital", "dementia services", "memory clinic", "mental health act", "geriatric", "elderly care". Citation chaining performed on all included sources. Targeted hand-searching of government portals, institutional websites, international agency repositories, and NGO publications (see **Panel B**). | None | Oct–Dec 2025; updated 16 Mar 2026 | N/A (targeted) |

**Note.** Google Scholar does not support full Boolean syntax or provide reliable total result counts. Multiple iterative searches were therefore conducted using the advanced search interface with combinations of terms; the first 20 pages of results were screened per query combination, as relevance declines substantially beyond this threshold.

**Panel B. Grey Literature and Supplementary Sources.**

| **Source Category** | **Specific Sources Searched** | **Identification Method** | **References Yielded** |
| --- | --- | --- | --- |
| Government portals and legislation | Pakistan Finance Division (Economic Surveys 2018–19 and 2023–24)  Sindh Laws portal (sindhlaws.gov.pk)  AJK Legislative Assembly (law.gok.pk)  Ministry of National Health Services (LHW Strategic Plan 2022–2028) | Targeted hand-searching of national and provincial government websites for mental health legislation, policy documents, and health expenditure data | [19, 52, 55, 58, 70] |
| Institutional and hospital websites | Aga Khan University Hospital, Karachi  King Edward Medical University, Lahore  Liaquat National Hospital, Karachi  Karwan-e-Hayat, Karachi  ConsidraCare Pakistan  SehatYab  College of Physicians and Surgeons Pakistan  MoCA Cognition (MoCA-Urdu test form) | Targeted review of hospital and organisational websites for service profiles, specialist clinic listings, and programme descriptions | [20, 24, 27, 28, 50, 53, 62, 63] |
| International agency reports | WHO Mental Health Atlas 2020 (Pakistan, Sri Lanka, Bangladesh country profiles)  WHO Mental Health Atlas 2024  WHO Global Dementia Observatory Reference Guide (2018)  WHO mhGAP Intervention Guide v2.0 (2016)  WHO Global Action Plan on Dementia 2017–2025  WHO World Mental Health Report (2022)  UN World Population Prospects 2024  World Bank health expenditure indicators (Pakistan) | Targeted searching of WHO, UN, and World Bank data repositories and publication catalogues | [1, 12, 13, 15, 17, 42, 48, 54, 66, 72] |
| NGO and research organisation publications | Alzheimer’s Disease International (Alzheimer’s Pakistan profile)  Alzheimer’s and Related Disorders Society of India (ARDSI)  Lanka Alzheimer Foundation (Sri Lanka dementia statistics)  Research Society of International Law, Pakistan (RSIL)  Oxford Policy Management (LHW evaluation) | Targeted searching of dementia-specific NGOs, mental health advocacy organisations, and policy research bodies | [8, 26, 36, 39, 59] |
| News media | Dawn (KP mental health institute; health scheme coverage)  The Nation (AKDN programme launch)  Daily Independent (AKDN programme launch) | Citation chaining from eligible sources; targeted searching for institutional inaugurations, policy announcements, and health coverage developments | [29, 30, 31, 67, 68] |
| Regional comparator sources (India, Bangladesh, Sri Lanka) | Ministry of Health and Family Welfare, India (NPHCE)  Global Health Network Asia (Bangladesh dementia data)  NIMH Sri Lanka (annual report) | Targeted searching for government health programme documentation in comparator countries | [34, 38, 40] |

**Panel C. Inclusion and Exclusion Criteria.**

| **Inclusion Criteria** | **Exclusion Criteria** |
| --- | --- |
| • Peer-reviewed articles reporting on dementia, geriatric psychiatry, cognitive screening, or mental health systems in Pakistan  • Government policy documents, mental health legislation, and official statistical reports from Pakistan (federal or provincial level)  • Grey literature from international agencies (WHO, World Bank, UN) relevant to Pakistan’s dementia burden, mental health system, or workforce  • Institutional service profiles, programme descriptions, and annual reports from psychiatric hospitals, memory clinics, or NGOs operating in Pakistan  • Comparative literature on dementia services or mental health systems in India, Bangladesh, and Sri Lanka (purposively selected regional comparators)  • Publications in English or Urdu  • No date restrictions applied | • Articles not relevant to Pakistan or the selected comparator countries (India, Bangladesh, Sri Lanka)  • Editorials, letters to the editor, or opinion pieces without substantive data, policy content, or service-level information  • Conference abstracts without sufficient methodological detail for data extraction  • Publications in languages other than English or Urdu  • Duplicate records (removed during deduplication using manual cross-referencing in Zotero) |

**Panel D. Screening Summary.**

| **Component** | **Details** |
| --- | --- |
| Databases searched | PubMed (263 results), Scopus (65 results), Google Scholar (7 iterative queries; first 20 pages screened per query) |
| Supplementary sources | Government portals, institutional websites, international agency repositories, NGO publications, news media, and citation chaining from eligible sources (see **Panel B**) |
| Initial search dates | 9–10 November 2025 (PubMed, Scopus, Google Scholar); supplementary searches conducted October to December 2025 |
| Search update | The search was updated in March 2026 to capture sources published during manuscript preparation; references 28, 62, 67, and 68 were identified during this update |
| Deduplication method | Manual cross-referencing in Zotero reference management software |
| Screening approach | Records were screened by title and abstract for relevance to the study objectives and inclusion criteria (Panel C). Full texts of potentially eligible sources were retrieved and assessed against the inclusion and exclusion criteria. Sources not meeting the criteria were excluded. |
| **Sources included in the final synthesis** | **72 (36 peer-reviewed articles and book chapters; 36 grey literature sources including government reports, legislation, institutional websites, NGO publications, international agency reports, and news articles)** |

**Note.** As this is an integrative review synthesising heterogeneous evidence sources rather than a systematic review, a formal PRISMA-style screening log was not maintained. The screening summary above reflects the search strategy and final source count. Full characteristics of all 72 included sources, including source type, geography, and appraisal, are provided in **Supplementary Table S1**.

**Supplementary Figure S1.** Flow of source identification, screening, and inclusion for the integrative review of provincial dementia service readiness in Pakistan.


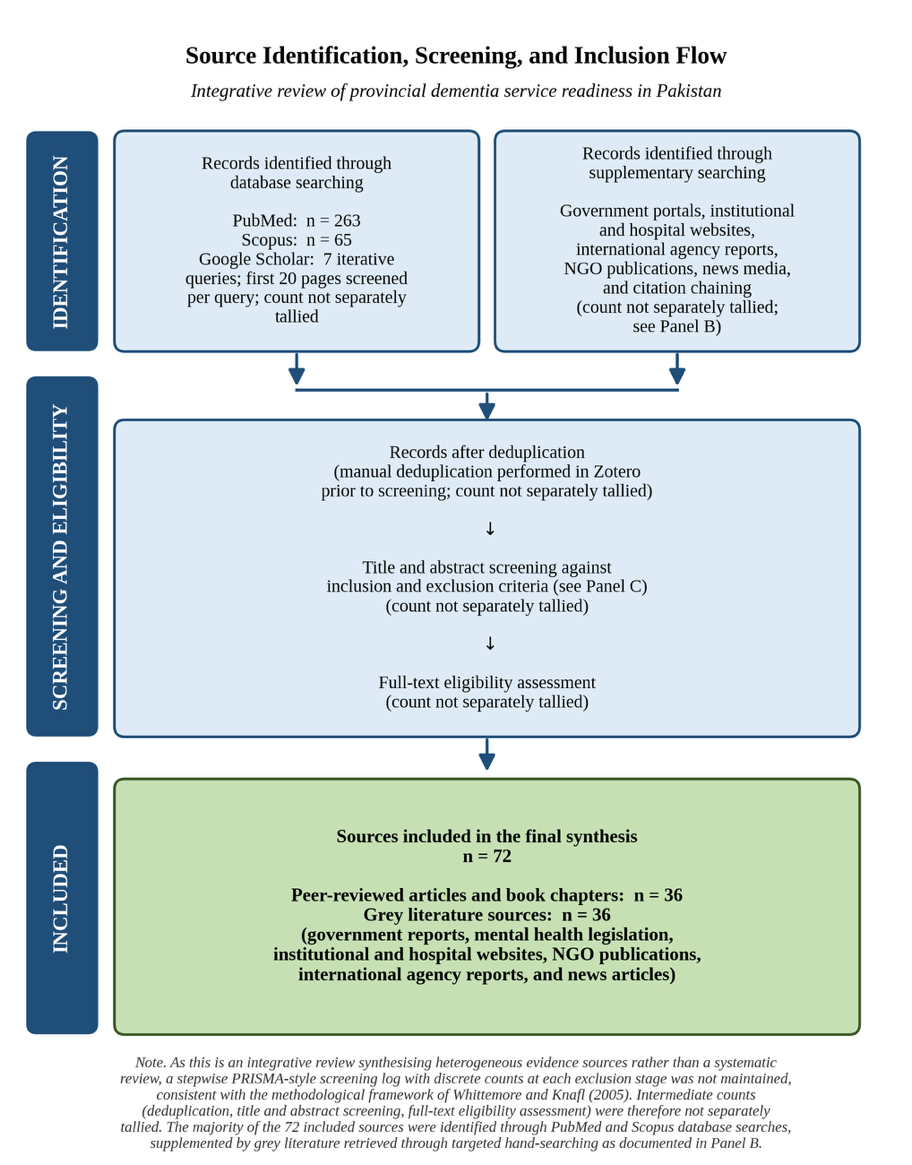


**Note**. As this is an integrative review synthesising heterogeneous evidence sources rather than a systematic review, a stepwise PRISMA-style screening log with discrete counts at each exclusion stage was not maintained, consistent with the methodological framework of Whittemore and Knafl (2005). The screening summary above and the corresponding flow in **Supplementary** **Figure S1** reflect the source-identification and inclusion counts that were tracked during the review. Full characteristics of all 72 included sources, including source type, geography, and appraisal, are provided in **Supplementary Table S1**.
